# Seasonal Shift in Physicochemical Factors Revealed the Ecological Variables that Modulate the Density of *Acinetobacter* Species in Freshwater Resources

**DOI:** 10.3390/ijerph17103606

**Published:** 2020-05-21

**Authors:** M. A. Adewoyin, A. I. Okoh

**Affiliations:** 1 SAMRC Microbial Water Quality Monitoring Centre, University of Fort Hare, Alice 5700, Eastern Cape, South Africa; aokoh@ufh.ac.za; 2 Applied and Environmental Microbiology Research Group, Department of Biochemistry and Microbiology, University of Fort Hare, Alice 5700, Eastern Cape, South Africa

**Keywords:** seasonal shift, physicochemical factors, freshwater resources, density, *Acinetobacter* species, correlation

## Abstract

Certain environmental variables are responsible for the survival of microorganisms in aquatic environments. The influence of these environmental factors in each season (winter, autumn, spring and summer) of the year can be used to track changes in a microbial population in freshwater resources. In this study, we assessed the effect of seasonal shifts in environmental variables including temperature, pH, total dissolved solids (TDS), total suspended solids (TSS), biochemical oxygen demand (BOD) and turbidity (TBS) among others on the density of *Acinetobacter* species in the Great Fish, Keiskamma and Tyhume rivers in the Eastern Cape Province, South Africa. Water samples and values of the environmental factors were taken from the rivers for 12 months. The density of presumptive *Acinetobacter* species was estimated from the culture of water samples on a CHROMagar selective medium, while the *Acinetobacter*-specific *recA* gene was targeted for the identification of *Acinetobacter* species using PCR assay. The multivariate relationship between seasons and changes in variables was created using PCA, while the effect of seasonal shifts in the environmental variables on the density of *Acinetobacter* species was evaluated using correlation test and topological graphs. Positive association patterns were observed between the seasons, environmental factors and the bacterial density in the rivers. In addition, temperature, TBS, TSS and BOD tended to influence the bacterial density more than other physicochemical factors in the rivers across the seasons. Of the total 1107 presumptive *Acinetobacter* species, 844 were confirmed as *Acinetobacter* species. Therefore, these findings suggested that the rivers contain *Acinetobacter* species that could be useful for basic and applied study in ecology or biotechnology, while their clinical relevance in causing diseases cannot be underestimated.

## 1. Introduction

Microbial communities in freshwater resources are extremely diverse [1,2] and they make major contributions in support of the functionality of the aquatic ecosystem upon which other higher organisms depend [3,4,5]. The survival of microorganisms in the aquatic environment depends on physicochemical variables such as temperature, pH, and turbidity etc. [6,7]. For instance, water turbidity is considered as an important indicator for the existence of high levels of pathogenic microorganisms, particularly bacteria, in water sources [8,9]. *Escherichia coli* in rivers have been reported to be very sensitive to the changes in physicochemical parameters because of pollution [10].

Some *Acinetobacter* species have been implicated in wound infection [11], bloodstream infection [12] and meningitis [13]. Additionally, selected strains of *Acinetobacter* species have shown ecological and economic importance such as in bioremediation of industrial effluents [14], degradation of oil spill [15] production of bioemulsifiers [16,17,18] and secretion of phytohormones [19].

Seasonal changes faced by the microbial communities inhabiting freshwater resources provide an opportunity to appraise the extent to which variation in the aquatic physicochemical variables affect the density and survival of the *Acinetobacter* species. One of the characteristics of the *Acinetobacter* species is the ability to survive in adverse environmental conditions [20,21]. The extremes of the physicochemical factors that influence the environment can be stressful for the survival and proliferation of microorganisms leading to ‘natural selection or survival of the fittest’. However, it depends on the ability of a particular microorganism to adapt to these physical changes in the environment.

Thus, the uncertainty that characterizes the shifts in weather conditions in South Africa was considered for the evaluation of the survival of the *Acinetobacter* species in the three major rivers in the Eastern Cape Province of South Africa. Understanding the response of *Acinetobacter* species in freshwater resources to changes in the environment during different seasons could serve as a tool to monitor the stress response of the microorganism when exposed to a sudden shift in environmental conditions. Therefore, given that some *Acinetobacter* species are potential waterborne pathogens [22] and some are useful bioremediation agents [14], this study was undertaken to evaluate the effect of seasonal peculiarities of the South African environmental conditions on the survival of *Acinetobacter* species in the freshwater resources.

## 2. Methods and Materials

### 2.1. Description of Sampling Sites

Water samples were collected from three rivers, namely the Great Fish, Keiskamma and Tyhume rivers in the Eastern Cape Province, South Africa, between April 2017, and March 2018. The Great Fish River is located in the Chris Hani District Municipality in the Eastern Cape Province and it is one of the major rivers used for irrigation and livestock farming in South Africa. This river is prone to agricultural and municipal runoffs and functions as the receiving stream of effluents from various wastewater treatment plants (WWTPs), particularly those located in urban residential districts such as Craddock. The Keiskamma and Tyhume rivers are located in the Amathole District Municipality in the Eastern Cape Province and are disclosed to different anthropogenic activities such as livestock drinking and irrigation agriculture in the rural and urban communities along the river courses. In summation, these rivers receive effluents from wastewater treatment plants (WWTPs) situated close to their banks. The river sampled and the corresponding sampling points are presented in Table 1. Different sampling points along the river courses were selected based on where humans and animal come into direct contact with them; for instance, points that are employed for fishing, drinking and swimming purposes and downstream of the WWTPs. Points, where irrigation water is discharged to the water bodies and proximity to hospital facilities, were also considered. The sampling period covers the four seasons (autumn, winter, spring, and summer) usually observed in South Africa.

### 2.2. Sample Collection

Water samples were collected aseptically in sterile 1 L glass bottles from the different sampling points along the river courses by midstream dipping of sample bottles at 25–30 cm down the water column, with the mouth tilting against the flow of the river. Between April 2017 and March 2018, 180 water samples were collected of which 30, 45, 30 and 75 water samples were collected in autumn, winter, spring and summer respectively. All the samples were labelled properly and transported to Applied and Environmental Microbiology Research Group (AEMREG) laboratory in an ice chest and processed within 6 h of collection.

### 2.3. Measurement of Physicochemical Variables of Freshwater Resources

The physicochemical parameters such as temperature (°C), pH, electrical conductivity (EC, µs/cm), salinity (PSU), total dissolved solids (TDS, (mg/dm^3^), total suspended solids (TSS (mg/dm^3^) and dissolved oxygen (DO, (mg/dm^3^) were measured on-site using a multi-parameter meter (Hanna, model HI 9828). Turbidity (TBS) (NTU) was determined using a turbidimeter (HACH, model 2100P). The five-day biochemical oxygen demand (BOD, (mg/dm^3^) of the samples was determined using a biochemical oxygen demand meter (HACH, HQ 40d) [23].

### 2.4. Isolation of Presumptive Acinetobacter Species

Bacterial density and presumptive *Acinetobacter* species in the water samples was determined by the membrane filtration technique [23]. Three volumes of 100 mL of each water sample were filtered through 0.45 μm (Ø 47 mm) pore sized filter paper under vacuum [23]. These membranes were aseptically placed on plates with *Acinetobacter* species selective medium, CHROMagar *Acinetobacter* plus selective supplement (CHROMagar, Paris, France), which was prepared according to the manufacturer’s instructions. The plates were incubated at 37 °C for 24 h and all colonies of microorganisms on plates were taken as bacterial density, while colonies showing red, a typical *Acinetobacter* species appearance on the CHROMagar medium were counted as presumptive *Acinetobacter* species (CFU/100 mL). The pure colonies of presumptive *Acinetobacter* species were stored in glycerol stocks at −80 °C for use.

### 2.5. DNA Extraction and Molecular Identification of Acinetobacter Species

DNA extraction from the bacterial isolates was carried out using the direct boiling method according to [24]. Polymerase chain reaction (PCR) assay was used for the amplification of *recA* gene by using *Acinetobacter* genus-specific primers for the identification of *Acinetobacter* species according to [25]. Thus, the population of *Acinetobacter* species in the initial presumptive *Acinetobacter* species density was confirmed.

### 2.6. Statistical Data Analysis

Seasonal changes in the physicochemical variables in the rivers were evaluated using Kruskal–Wallis ANOVA (H-test). The Kruskal–Wallis *H*-test was employed for the statistical analysis because the months that make the seasons are not parametric, while multiple comparison post hoc was used to determine the significant values at *p* < 0.05. Principal component analysis (PCA) (Biplots) was employed to define the seasonal changes in the physicochemical variables in each river using PAST (v 4.0, Øyvind Hammer, Natural History Museum, University of Oslo, Norway). The correlation between the physicochemical variables and the density of presumptive *Acinetobacter* species in the three rivers across the four seasons was measured by correlation test in Statistica software version 13. Coefficient of correlation (*r*) between physicochemical variables and presumptive *Acinetobacter* species was determined using Pearson’s correlation (*p*-value < 0.05), while further validation of the relationship between physicochemical variables and presumptive *Acinetobacter* species was performed using Kohonen analysis (*p* < 0.05) trial version of XLstat2018 (Excel Addinsoft, Paris, France).

## 3. Results

### 3.1. Physicochemical Parameters Vary with Seasons

The physicochemical variables of the freshwater resources were recorded within the four seasons of the year in South Africa. Table 2 presents the various physicochemical parameters in the Great Fish, Keiskamma and Tyhume rivers respectively. Certain physicochemical properties of the freshwater resources varied markedly with the seasons, while others remained relatively the same.

The pH values of the water samples collected from the Great Fish river remained within the alkaline range (8.0–8.2), while the Keiskamma and Tyhume rivers were relatively neutral (7.2–7.9). However, there was significant (*p* < 0.05) variation in pH across the seasons in the Great Fish (*H* = 17.43; *p* = 0.0006), Keiskamma (*H* = 29.65; *p* = 0.0000) and Tyhume (*H* = 40.27; *p* = 0.0000) rivers.

The temperature increased from winter to summer (winter<autumn<spring<summer), and the Great Fish was the hottest among the rivers studied. Summer came with a significant (*p* < 0.05) increase in temperature, compared to other seasons when evaluated in the Great Fish (*H* = 140.16; *p* = 0.000), Keiskamma (*H* = 152.31; *p* = 0.0000) and Tyhume (*H* = 95.53; *p* = 0.0000) rivers.

An average EC and TDS of the water samples from the three rivers were significantly (*p* < 0.05) lower during summer compared to other seasons. In both the Great Fish and Keiskamma rivers, EC during the autumn, winter and spring was not significantly different (*p* < 0.05) from each other, but there was a significant decrease (*p* < 0.05) in EC during the summertime. The EC in Tyhume was similarly low in summer, but no statistical difference (*p* < 0.05) was observed. However, there was significant shift in EC in Great Fish (*H* = 72.40; *p* = 0.000) and Keiskamma (*H* = 46.18; *p* = 0.0000) but not in Tyhume (*H* = 0.92; *p* = 0.8211) across seasons. Comparable to EC, TDS was significantly lower (*p* < 0.05) in summer compared to other seasons in all the rivers evaluated in this study. Nonetheless, the change in TDS was statistically significant across season in Great Fish (*H* = 71.35; *p* = 0.0000), Keiskamma (*H* = 32.10; *p* = 0.0000) but not in Tyhume (*H* = 0.96; *p* = 0.8115).

Additionally, there was substantial change in salinity in each of the river across the seasons in both Great Fish (*H* = 71.99; *p* = 0.0000) and Keiskamma (*H* = 48.87; *p* = 0.0000) compared to Tyhume (*H* = 2.07; *p* = 0.5585).

TSS increased from spring to summer (spring<autumn<winter<summer). TSS in summer was significantly higher (*p* < 0.05) than other seasons. However, change in TSS was statistically significant across the season in Great Fish (*H* = 142.24; *p* = 0.0000), Keiskamma (*H* = 33.96; *p* = 0.0000) but not in Tyhume (*H* = 6.10; *p* = 0.1068). Similarly, while TBS increased from spring to summer (spring<winter<autumn<summer) in all the rivers, there was significant (*p* < 0.05) increase in TBS in the summertime compared to autumn, winter and spring. However, TBS in the Great Fish (*H* = 146.31; *p* = 0.000) was statistically significant compared to both Keiskamma (*H* = 31.43; *p* = 0.0000) and Tyhume (*H* = 10.95; *p* = 0.0120) rivers in all the seasons.

Additionally, there was no significant change (*p* < 0.0%) in DO between autumn, winter and spring in each of the rivers. However, DO in the summertime was lower than autumn, winter and spring in each of the rivers. The seasonal change in DO was statistically significant in the Great Fish (*H* = 137.42; *p* = 0.0000), Keiskamma (*H* = 106.9079; *p* = 0.000) and Tyhume (*H* = 94.73601; *p* = 0.000) rivers in all the seasons.

BOD in the Great Fish river was highest in winter even though there was no statistical difference (*p* < 0.05) in the values across the seasons. In the Keiskamma River, BOD was notably high in spring compared to autumn and winter, but no significant difference (*p* < 0.05) between the BOD was recorded in the spring and summer seasons. In the Tyhume river, BOD in spring was similarly high compared to the other seasons studied. Notwithstanding, there was statistical significance of BOD in the Great Fish (*H* = 41.35323; *p* = 0.0000), Keiskamma (*H* = 34.97011; *p* = 0.000) and Tyhume (*H* = 26.08914; *p* = 0.000) rivers in all the seasons.

Therefore, most of the environmental variables changed across the seasonal gradients, indicating contrasting aquatic environments among the rivers studied.

Principal component analysis was employed to create a multivariate association between the physicochemical parameters and the seasons in each of the three rivers studied.

**Figure 1:**
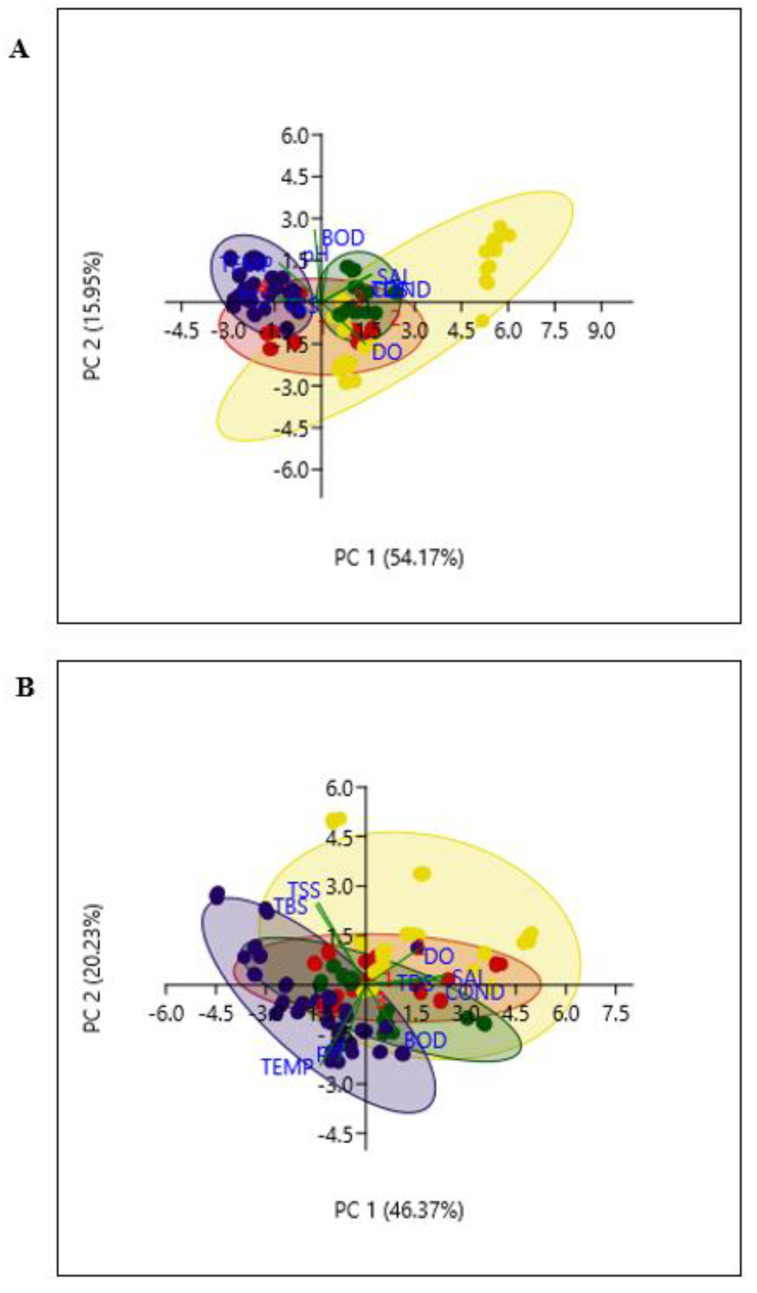

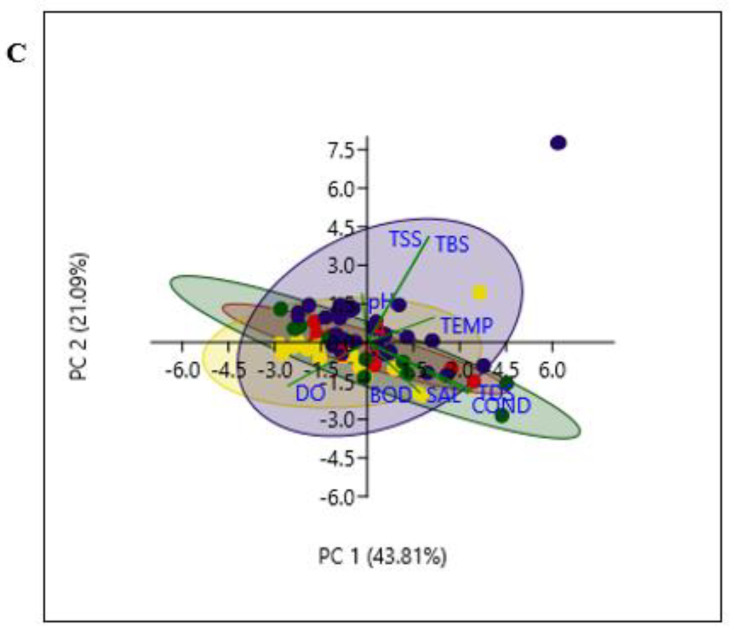
Seasonal changes in the physicochemical variables in (**A**) Great Fish, (**B**) Keiskamma and (**C**) Tyhume, rivers. Principal component analysis of the physicochemical variables in each river across the four seasons: (1) Autumn (●), (2) Winter (●), (3) Spring (●) and (4) Summer (●). The lengths and directions of the Biplots vectors explain the association between the variables and the seasons. Measurement of physicochemical variables of freshwater resources was done in three replicates (n = 3).

Of the nine principal components of the analysis, the first two components (PC1 and PC2) were chosen for further analysis in all the rivers using the scree plot principle of eigenvalues. The two PCs explain 70.11%, 66.63% and 64.90% of the observed variation (*p* < 0.05) in the Great Fish, Keiskamma and Tyhume rivers respectively. However, for a convenient interpretation of the analysis, variables with loadings (coefficients) as indicated below were retained to establish the influence of each variable on the component. Biplot of PC1 versus PC2 in each of Great Fish, Keiskamma and Tyhume rivers are presented in Figure 1A–C respectively.

In the Great Fish river, PC1 explains 54.17% of the observed variability. PC1 had large positive associations with EC (*r* = 0.41), TDS (*r* = 0.41), salinity (*r* = 0.41) and DO (*r* = 0.36); and negative association with temperature (*r* = −0.35), TSS (*r* = −0.35) and TBS (*r* = −0.35). Both pH (*r* = 0.05) and BOD (*r* = -0.05) had a weak positive and negative correlation with PC1. PC2 (15.95%) had a large positive correlation with BOD (*r* = 0.66) and temperature (*r* = 0.36) and negative correlation with DO (*r* = −0.38), while association with other parameters was weak at ≥95% confidence level.

Similarly, PC1 in the Keiskamma river accounts for 46.4% of the observed variability showing large positive associations with EC (*r* = 0.46), TDS (*r* = 0.45) and salinity (*r* = 0.46), while pH (*r* = −0.16), temperature (*r* = −0.26), TSS (*r* = −0.28) and TBS (*r* = −0.29) had weak negative associations. However, DO (*r* = 0.30) and BOD (*r* = 0.18) had a weak positive association with PC1. PC2 (20.23%) had a large positive correlation with TSS (*r* = 0.48) and TBS (*r* = 0.47) and large negative correlation with pH (*r* = −0.45) and temperature (*r* = −0.47) at ≥95% confidence level.

PC1 in the Tyhume river accounts for 43.81% of the observed variability with large positive associations with EC (*r* = 0.46), TDS (*r* = 0.44), and salinity (*r* = 0.46), while pH (*r* = −0.17), temperature (*r* = −0.30), TSS (*r* = −0.28) and TBS (*r* = −0.29) had a weak negative association with PC1. PC2 (21.09%) had a large positive correlation TSS (*r* = 0.57) and TBS (*r* = 0.56) at ≥95% confidence level. Correlation between DO and PC1 was *r* = 0.32, while both PC1 and PC2 had a negative association with temperature (*r* = −0.30; *r* = −0.37), BOD (*r* = −0.06; *r* = −0.22) and pH (*r* = −0.17; *r* = −0.41).

Generally, factors having higher coefficients tend to influence the component and seasonal shifts in the physicochemical parameters of the three rivers (Table 2). Thus, EC, salinity and TDS had a substantial influence on PC1, while the second principal component (PC2) was influenced by temperature, TSS and TBS, with larger negative or positive loadings than PC1.

Based on the above view, the eigenvectors in the two PCs showed that temperature, TBS and TSS were strongly associated with the summer season in all the rivers. DO was partly connected with autumn, winter and spring in all the rivers (Figure 1; Table 2). Additionally, the multivariate association between the environmental variable and the seasons indicated that the EC, SAL and TDS were connected to autumn, winter and spring. The ellipsoidal system of 95% confidence level that clustered the variable in each season group helped to reliably visualize the multivariate distribution of the physicochemical parameters in the biplot. The marked differences in the Biplot and ellipses of the rivers showed that there were seasonal shifts in the physicochemical variables of the freshwater resources, depending on the river analyzed.

### 3.2. Density of Acinetobacter Species in the Freshwater Resources

**Figure 2:**
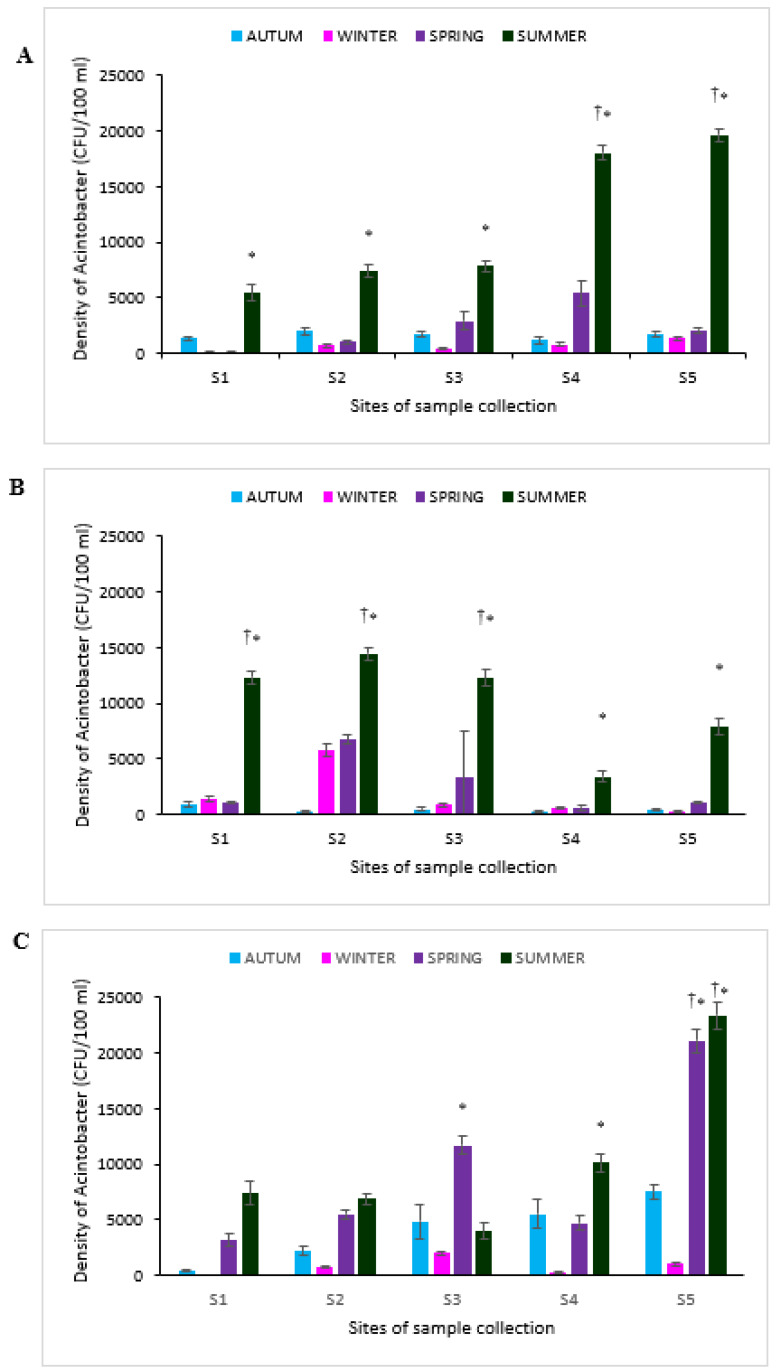
Density of the presumptive *Acinetobacter* species in (**A**) Great Fish (**B**) Keiskamma and (**C**) Tyhume rivers across the four seasons. Three replicates of each site were taken and analyzed. The column of the graph shows the mean, while the error bars indicate the standard deviations. Four seasons were evaluated: Autumn—April and May; Winter—June, July, and August; Spring—September and October; Summer—November, December, January, February, and March). * Indicates statistically significant (*p* ≤ 0.05) density of *Acinetobacter* species recovered in a specific season in comparison to other seasons for a given river. † Indicates statistically significant (*p* ≤ 0.05) density of *Acinetobacter* species recovered in one site compared to other sites. S1–S5 stands for site 1 to site 5.

Figure 2A–C present the density of presumptive *Acinetobacter* species recovered from the five sampling sites of the Great Fish, Keiskamma and Tyhume rivers respectively, in accordance to the four seasons of the year. Supplementary Figures S1 and S2 show the culture plate and pure culture of presumptive *Acinetobacter* species. On the plate (Figure S1), different colonies of bacteria were observed showing blue and red, which suggested that the CHROMagar medium might contain ingredients that could engender the growth of other microorganisms such as *E. coli* (blue appearance).

However, an average density (counts) of presumptive *Acinetobacter* species recovered during the autumn, winter, spring and summer was 1602 ± 257.38, 684.89 ± 114.91, 2312.67 ± 477.36 and 11655.33 ± 608.70 (CFU/100 mL) in the Great Fish River. Presumptive *Acinetobacter* species in Keiskamma were 460.97 ± 118, 1775.78 ± 223.15, 2577.67 ± 1006.99 and 10040.53 ± 598.48 (CFU/100 mL), while 4096 ± 805.45, 830 ± 111.14, 9216.67 ± 689.74 and 10331.07 ± 862.83 (CFU/100 mL) were recorded in Tyhume. In all the rivers, there was significant (*p* < 0.05) recovery of presumptive *Acinetobacter* species during the summer season compared to other seasons in this study. However, in the Tyhume River, the density of presumptive *Acinetobacter* species recovered during spring and summer seasons were significantly higher (*p* < 0.05) than autumn and winter (Figure 2C). Additionally, while presumptive *Acinetobacter* species recovered during the winter and autumn was significantly lower (*p* < 0.05) than the spring and summer seasons in the Great Fish river, recovery of the *Acinetobacter* species in the Keiskamma river during the summer seasons were significantly higher (*p* < 0.05) than other seasons. Nonetheless, a substantial number of *Acinetobacter* species were recovered at sampling point 2 (Keiskamma river) during the winter and spring compared to other sampling sites and autumn (Figure 2B).

**Figure 3:**
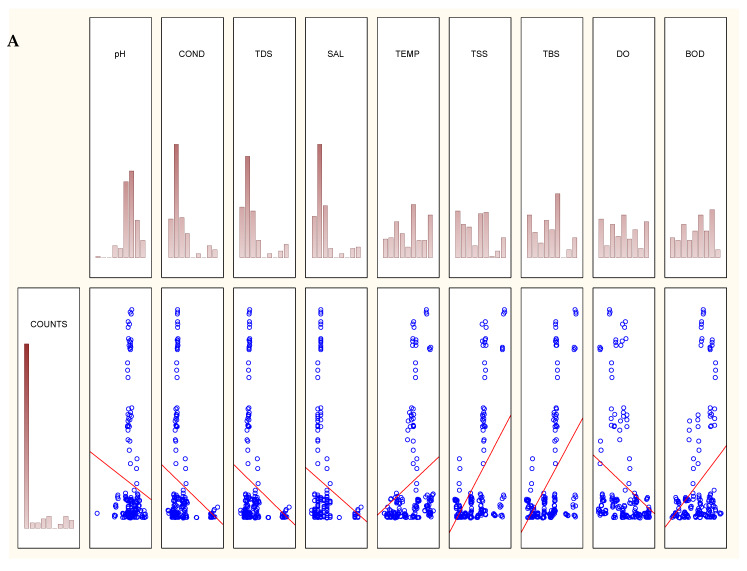

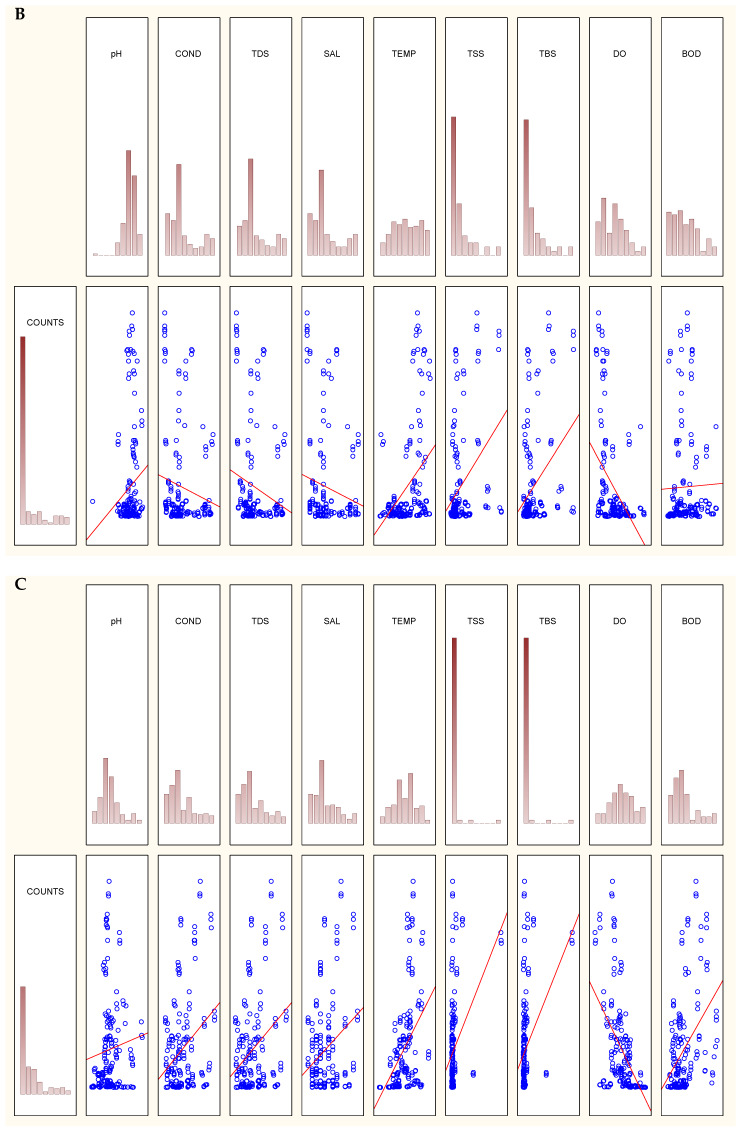
Correlation test for the relationship between the counts of presumptive *Acinetobacter* species and physicochemical variables. Counts histogram (**A**–**C**) correspond to Great fish, Keiskamma and Tyhume rivers, respectively. The trend line in the correlation charts indicates the nature of the correlation – positive or negative.

To further evaluate the effect of changes in the environmental variables on the density of *Acinetobacter* species in more details, we examined whether both the physicochemical variables and the density have any correlation using correlation test and topological graphs (Kohonen analysis). Table 3 presents the coefficient of correlation (*r*) and statistical significance (*p*-values) of the correlation test, while Figure 3A–C show the graphical representations of the relationship.

The density of the presumptive *Acinetobacter* species in the river varied significantly (Pearson’s; *p* < 0.05) as seasonal change affected physicochemical variables (Table 3; Figure 3). The positive and significant correlation between the physicochemical variables and the bacterial density revealed that temperature (*r* = 0.2416; *p* = 0.001), TSS (*r* = 0.4639; *p* = 0.000), TBS (*r* = 0.4483; *p* < 0.000) and BOD (*r* = 0.3079; *p* = 0.000) were responsible for the bacterial density in the Great Fish River. In the Keiskamma River, pH (*r* = 0.1434; 0.055) temperature (*r* = 0.3816; *p* = 0.000), TSS (*r* = 0.3572; *p* = 0.000), TBS (*r* = 0.3512; *p* = 0.000) and BOD (*r* = 0.0234; *p* < 0.755) were the driving factors for the density of *Acinetobacter* species. While all these factors influenced the density of *Acinetobacter* species in Tyhume, DO did not correlate with the density. Further consideration of PCA in Section 3.1 shows that variables that correlated with PC2 are the main drivers of the density of *Acinetobacter* species in the rivers.

**Figure 4:**
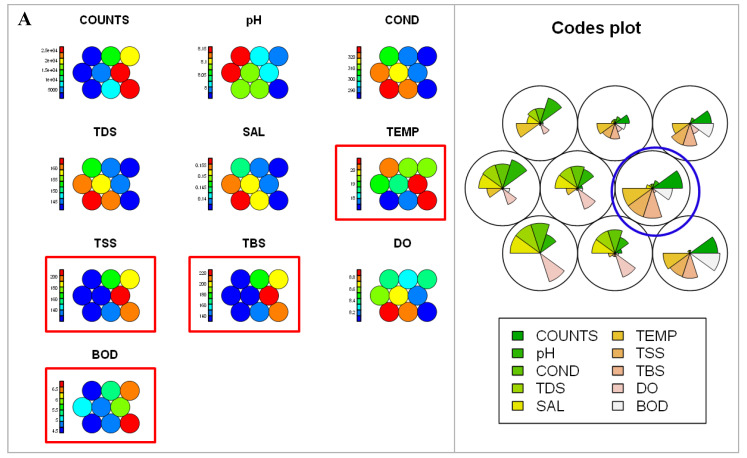

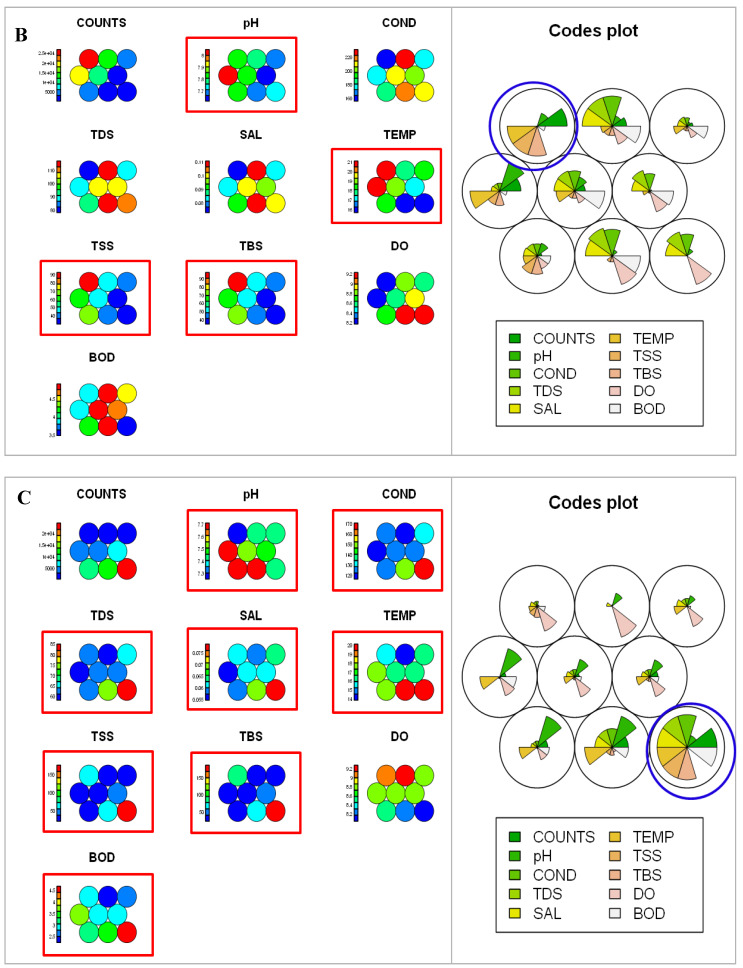
Kohonen topological graphs (self-organising maps - SOMs and Codes plot) showing a visual relationship between the physicochemical variables and *Acinetobacter* species density in: (**A**) Great Fish river: temperature, TSS, TBS and BOD were positively correlated with *Acinetobacter* species density; (**B**) Keiskamma river: temperature, TSS and TBS positively correlated with *Acinetobacter* species density; (**C**) Tyhume river: all physicochemical variables except DO and pH are correlated with *Acinetobacter* species density. The ruler shows the range of the variable, where RED is highest and blues the least values. SOMs and codes plots of the physicochemical variables that significantly contributed to the bacterial density in each of the rivers are captured in rectangle and circular shapes.

Comparable with the correlation/association test, Kohonen self-organizing maps (SOMs) and codes plots were used to connect the bacterial counts and physicochemical variables in the various rivers studied. The average distance to the closest codebook vector was made with 100 iterations (training progress) for uniformity among the rivers. Figure 4A–C presents the hexagonal topological SOMs (clusters) and codes plots of the relationships between the physicochemical variables and the density of presumptive *Acinetobacter* species in the Great Fish, Keiskamma and Tyhume rivers respectively. SOMs and codes plots of the physicochemical variables showing significant (*p* < 0.05) effect on the density of *Acinetobacter* species in each of the rivers are captured in a rectangle and circular shapes respectively.

The SOMs visually indicated that temperature, TSS, TBS and BOD are responsible for the bacterial counts in the Great Fish River, meanwhile, pH, temperature, TSS and TBS were positively correlated with the bacterial density in the Keiskamma River. Likewise, in the Tyhume River, both pH and DO did not have any relationship with the density of presumptive *Acinetobacter* species.

Even so, depending on the magnitude of each variable in the weight vector, the codes plot further confirmed the effect of each physicochemical variable on the density of presumptive *Acinetobacter* species in the river across the season. As such, code plots indicated that the bacterial density in Tyhume River correlates with all the physicochemical variables except DO. Although, correlation-association test showed the relationships between the physicochemical variables and bacteria counts. However, both SOMs and codes plots showed better pieces of evidence about the levels of the relationship between the bacterial density and physicochemical variables.

### 3.3. Confirmation of Acinetobacter Species from Presumptive Species

As shown in Figure S1, the pure culture of bacteria colonies with an appearance of red colour (typical colour of *Acinetobacter* species on CHROMagar) was 1107 presumptive *Acinetobacter* species of which 370, 309 and 428 were isolated from Great Fish, Kieskamma and Tyhume respectively.

Ultimately, after screening with PCR assay targeting *Acinetobacter*-specific *recA* gene (Figure S2), 844 confirmed *Acinetobacter* species were recovered of which 285, 219 and 340 were isolated from the Great Fish, Keiskemma and Tyhume rivers respectively.

## 4. Discussion

In this study, we evaluated the influence of seasonal shifts in the physicochemical variables on the survival of *Acinetobacter* species in the three freshwater resources in the Eastern Cape Province of South Africa.

The relationships between physicochemical variables and seasons were assessed using PCA; while self-organized maps (SOMs) and Codes plots (Kohonen topological graphs) were advanced to measuring the effect of physicochemical variables and density of *Acinetobacter* species. The limitations of PCA have been observed when it comes to identifying the relationship between the density of bacteria and environmental factors [27,28]. However, to a certain extent, PC2 identified factors that significantly contributed to the density of *Acinetobacter* species in the rivers, while PC1 indicated factors that contributed less.

The variations in the seasons were observed to influence the gradient of the physicochemical variables and the density of presumptive *Acinetobacter* species within the freshwater resources, even though the relationship between seasonal shifts in physicochemical variables and the density of presumptive *Acinetobacter* species were river-dependent. Anthropogenic activities (Table 1) in the area studied and changes in the seasons could have mediated changes in physicochemical variables and the density of presumptive *Acinetobacter* species. For example, it was stated in Table 1 that the areas where water samples were collected were characterized by farming, fishing, run-off and discharge of effluents from WWPT, etc.

The average pH of the rivers sampled fell within a neutral and slightly alkaline range in agreement with other reports [7,29,30]. However, the pH of Great Fish river water slightly exceeded the required limit (6.5–8.0) [31] for domestic and agricultural activities during autumn and spring seasons compared to other rivers. This might be linked with farming; exposure to wastewater effluent from the WWPT (GF4) and animal rearing. Increase in pH might also arise from the disposal of highly alkaline domestic wastewater (washing of plates and laundry) as well as traditional ritual washing at GF5 along the river courses. During the spring, agricultural activities usually increase and application of alkaline fertilizers could raise the pH of the water body due to inflow from farmland whenever rainfalls [32]. Extremity of pH (acidity or alkalinity) is an indication of poor water quality [33], which could alter biochemical operations in freshwater resources. Thus, the effect of slight changes in pH on the bacteria density was only noticeable in Keiskamma and Tyhume rivers, where pH values look favourable for the survival of organisms, (Table 2). Although, evaluation of the dataset using Kohonen topological graph (SOMs and Codes plots) revealed that the correlation between pH and bacterial density in Keiskamma and Tyhume rivers was not statistically significant (Figure 4). As such, the stability of pH around neutral in freshwater resources is nonetheless important for the survival of living organisms.

Seasonal change significantly influenced temperature, TBS and TSS gradients, especially in the Great Fish and Keiskamma rivers (Table 2). Naturally, environmental temperature tends to influence TBS and TSS alike. Temperature is normally at the extreme during the summer in South Africa, while high turbidity could be associated with dryness and lightness of nonliving matter such as silt and sand particles, which are easily stirred up by the wind in summer. The average turbidity values of the water samples in Table 2 showed that the Great Fish River was the most turbid, although all the rivers sampled significantly exceeded the standard limit [31]. *Acinetobacter* species have been observed to grow at a temperature between 20 and 44 °C [34], which might have an enormous influence on the proliferation of the organism in the Great Fish river compared to other rivers during the summer season.

For instance, Great Fish had the highest temperature, TSS and TBS, which corresponded with the density of presumptive *Acinetobacter* species. Besides, with the increase in temperature, TSS and TBS during the summer [35], more nutrients in the form of organic matters in the water [36,37] would be available for the microorganism in the rivers owing to instream overflow from soil surface, especially from the nearby dumpsite. Additionally, there could be an increase in retention of TSS and storage of nutrients, which could contribute to the growth of organism during the summer.

Water TBS is an important indicator for the presence of pathogenic microorganisms such as *Acinetobacter* species and other disease-related bacteria [38]. The increase in mean values of the TBS in this study could be an indication of silt content of the water, which in turn might lead to an increase in the number of pathogenic microbes. Additionally, occurrence of higher indicator bacterial numbers in any water sample could be linked to higher turbidity levels [39].

Comparably, other seasons, especially spring that is characterized with rainfall is not so because erosion would not allow the availability of nutrients to organisms. Thus, seasonal variation of the density of *Acinetobacter* species suggested that temperature, TSS and TBS favourably supported the optimal growth of the microorganism, which occurred in the summer compared to autumn, winter and spring.

The source of EC, salinity and TDS of freshwater resource (Table 2) could be attributed to solid features or rocks found in the geographical location, which eventually dictates the composition of the soil and water [40]. Other major factors that drive EC in rivers could be washing where detergent or anionic/cationic surfactant are released into water [41,42] and thus increase conductivity. However, ECs were below the required standard for drinking water as presented in Table 2. While, EC, SAL and TDS of freshwater change from season to season, they were not significantly involved in the density of *Acinetobacter* species in each river. High levels of EC in freshwater resources can be dangerous to the existence of their aquatic life; increased salinity in freshwater could result in the smothering of the river bottom, particularly in the deep rivers [43]. Thus, the effect of seasonal shift on these parameters was not significant on the density of *Acinetobacter* species in the rivers.

The BODs of the water sources were within the required limit of 3–6 mg/dm^3^ respectively for water [31,44]. The variation in seasons significantly affect changes in BOD, though is an indicator of the health of living organisms in an aquatic environment [45]. Adjustment in BOD could also be influenced by anthropogenic activities such as agricultural practice and discharge of domestic wastewater, sewage, animal and human wastes, which are commonly practised in the area (Table 1). The metabolic activities of most organisms are aerobically controlled, and therefore, oxygen is an indispensable element for the survival of organisms at all times. 

DO of the freshwaters resources were within the permissible limit of ≥5 mg/dm3 (Table 1) for drinking water and South African freshwater quality [31], developed a model that simulated the seasonal cycle of DO in the Chesapeake Bay during the summer [46]. From their work, water column respiration contributed to about 74% of the total biological consumption and sediment oxygen demand accounted for about 26% during the summer, which was why DO was lower in summer than another season in this work. The level of DO in freshwater could be influenced by metabolic processes, temperature, pollution and organic matter level as well as the respiration of microorganisms that are involved in the decomposition of the pollutants [47]. Linking the anthropogenic activities listed in Table 1 for the areas where water samples were collected, the tendency for river pollution was very high. Therefore, the significant reduction in DO during the summer is adduced to this observation. As such, none of the correlation tests associated it with the density of *Acinetobacter* species in the Great Fish, Keiskamma and Tyhume rivers (Table 2).

From the forging, bacteria belonging to the genus *Acinetobacter* from presumptive species (CHROMagar culture) were identified using the molecular confirmatory test of by targeting *recA* gene [25,48], which has described *recA* gene as a reliable method for identification of *Acinetobacter* [25].

## 5. Conclusions

This study of the effect of seasonal shifts in environmental factors on the survival of *Acinetobacter* species has created an opportunity to detect statistical significance in the relationship between the density of presumptive *Acinetobacter* species and physicochemical variables in the Great Fish, Keiskamma and Tyhume rivers. From the various analyses in this study, temperature, TSS, TBS and BOD were the main but not the only factors that were influencing the density of *Acinetobacter* species in the various rivers. Overall, seasonal changes in the identified variables tended to control the quantity of presumptive *Acinetobacter* species recovered from each river. The response of *Acinetobacter* species to the shifts in environmental conditions is a semblance of a model to predict the nature of *Acinetobacter* species in the freshwater resources. As such, this study revealed that the three rivers that were investigated contain *Acinetobacter* species that might be useful for basic and applied study in ecology, microbiology and biotechnology, while their clinical relevance in causing diseases cannot be overemphasized. Thus, these rivers deserve further evaluation using advanced methods such as metagenomics.

## Figures and Tables

**Table 1:** Description of different sampling sites on the Great Fish, Keiskamma and Tyhume rivers.

Rivers	Site Codes	Site Name	Major Human Activities	Coordinates
Great Fish	GF1	Craddock upstream 1	Irrigation farming, fishing	S 32°05.902′ E 025°35.784′
	GF2	Craddock upstream 2	Fishing, farming, animal rearing	S 32°19.169′ E 025°36.823′
	GF3	Craddock township 1	Fishing, swimming, animal rearing, domestic purposes	S 32°10.256′ E 025°36.831′
	GF4	Craddock township 2	Downstream of Craddock WWTP, fishing, swimming, animal rearing, refuse dumping	S 32°11.322′ E 025°37.846′
	GF5	Craddock location	Fishing, swimming, traditional ritual washing, refuse dumping	S 32°11.527′ E 025°39.616′
Keiskamma	KE1	Keiskammahoek upstream	Farming, animal rearing	S 32°38.427′ E 027°11.436′
	KE2	Keiskammahoek township 1	Domestic use, car washing, domestic waste pipe leakages, animal rearing, community runoff	S 32°40.538′ E 027°08.477′
	KE3	Keiskammahoek township 2	Refuse dumping, community runoff, water pipe leakages	32°41.271′ E 027°09.127′
	KE4	Keiskammahoek downstream	Animal rearing, Farming, downstream of Sandile Dam,	S 32°44.292′ E 027°05.895′
	KE5	Sandile community	Downstream of Sandile WWTP, animal rearing, farming, vehicles crossing	S 32°45.579′ E 027°04.110′
Tyhume	TY1	Tyhume source	Tourism, swimming	S 32°36.683′ E 022°57.612′
	TY2	Kayaletu village	Domestic use, animal rearing, farming, community runoff	S 32°38.374′ E 026°56.163′
	TY3	Bin field Dam	Fishing, recreational purposes, farming, animal drinking	S 32°40.980′ E 026°54.080′
	TY4	Melani village	Swimming, domestic use, fishing, animal rearing	S 32°43.223′ E 026°51.646′
	TY5	Alice town	Fishing, construction purposes, farming, downstream of hospital waste discharge, UFH wastes discharge, animal rearing	S 32°46.920′ E 026°50.796′

**Table 2:** Physicochemical parameters of the Great Fish, Keiskemma and Tyhume rivers across the four seasons.

Parameter	pH	Temperature (°C)	EC (µS/cm)	TDS (mg/dm^3^)	SAL (PSU)	TSS (mg/dm^3^)	Turbidity (NTU)	DO (mg/dm^3^)	BOD (mg/dm^3^)
Great Fish River									
Autumn	8.2 ± 0.3	15.8 ± 1.1	299 ± 39.4	149 ± 19.7	0.14 ± 0.01	57 ± 49.5	168 ± 49.1	9.0 ± 0.4	3.1 ± 1.6
Winter	8.0 ± 0.4	12.7 ± 1.4	369 ± 113.5	184 ± 56.8	0.18 ± 0.1	85.6 ± 24.0	96 ± 27.4	9.9 ± 0.4	4.9 ± 2.5
Spring	8.2 ± 0.2	20.3 ± 0.7	339 ± 28.4	169 ± 14.1	0.16 ± 0.0	44.3 ± 8.9	48 ± 10.5	8.5 ± 0.4	3.9 ± 2.1
Summer	8.0 ± 0.4	22.3 ± 2.6	274 ± 18.2	137 ± 8.9	0.13 ± 0.01	99.4 ± 43.6	214 ± 45.9	7.8 ± 0.5	3.7 ± 0.6
Keiskemma River									
Autumn	7.7 ± 0.3	14.5 ± 1.9	247 ± 101.8	123 ± 50.8	0.12 ± 0.1	31 ± 24.8	36 ± 29.1	9.1 ± 0.4	3.0 ± 2.0
Winter	7.5 ± 0.4	11.0 ± 1.9	285 ± 115.3	143 ± 57.7	0.14 ± 0.1	39 ± 59.3	42 ± 58.0	9.8 ± 0.6	3.2 ± 2.2
Spring	7.7 ± 0.3	16.1 ± 1.3	216 ± 102.2	108 ± 51.4	0.10 ± 0.1	27 ± 15.1	31 ± 17.6	9.2 ± 0.7	6.0 ± 2.6
Summer	7.9 ± 0.5	21.4 ± 1.9	153.2 ± 63.7	86 ± 33.0	0.07 ± 0.0	55.6 ± 48.4	61 ± 49.6	8.3 ± 0.5	4.5 ± 1.9
Tyhume River									
Autumn	7.5 ± 0.3	15.4 ± 2.8	141 ± 62.7	71 ± 31.3	0.07 ± 0.0	30 ± 24.2	35 ± 27.6	8.9 ± 0.6	2.4 ± 0.8
Winter	7.2 ± 0.5	11.3 ± 3.0	129 ± 56.9	65 ± 28.4	0.06 ± 0.0	51 ± 133.3	57 ± 151.8	9.8 ± 0.6	2.0 ± 0.8
Spring	7.3 ± 0.5	16.8 ± 3.5	141 ± 85.6	70 ± 42.7	0.07 ± 0.0	30 ± 29.4	35 ± 31.0	8.9 ± 0.8	4.2 ± 2.6
Summer	7.7 ± 0.5	20.2 ± 4.1	125 ± 50.8	62 ± 25.6	0.06 ± 0.0	89.6 ± 243.2	96 ± 255.9	8.2 ± 0.7	3.4 ± 1.7
Regulation [26]	5.5–9.5	≤35	7000–15000	450	-	-	<5	<5	3–6

EC = electrical conductivity; TDS = total dissolved solid; SAL = salinity; TSS = total suspended solid; DO = dissolved oxygen; BOD = biological oxygen demand.

**Table 3:** Correlation between the density of presumptive *Acinetobacter* species and the physicochemical variables in the three rivers studied.

Physicochemical Variables	Great Fish		Keiskamma		Tyhume	
	*r*-Value	*p*-Value	*r*-Value	*p*-Value	*r*-Value	*p*-Value
pH	−0.0915	0.222	0.1434	0.055	0.0875	0.243
EC	−0.1886	0.011	−0.1367	0.067	0.3134	0.00
TDS	−0.1901	0.011	−0.1772	0.017	0.3133	0.000
SAL	−0.1790	0.016	−0.1430	0.055	0.2709	0.000
TEMP	0.2416	0.001	0.3816	0.000	0.4228	0.000
TSS	0.4639	0.000	0.3572	0.000	0.3633	0.000
TBS	0.4483	0.000	0.3512	0.000	0.3581	0.000
DO	−0.2644	0.000	−0.4084	0.000	−0.4975	0.000
BOD	0.3079	0.000	0.0234	0.755	0.4202	0.000

## Supplementary Materials

The following are available online at https://www.mdpi.com/1660-4601/17/10/3606/s1, Figure S1: Pure culture of presumptive Acinetobacter species streaked on CHROMagar selective medium for the genus Acinetobacter: (**A**) Great fish (**B**) Keiskamma and (**C**) Tyhume Rivers. Seasons: Autumn—April and May; Winter—June, July, and August; Spring—September and October; Summer—November, December, January, February, and March. Figure S2: Gel electrophoresis of confirmed *Acinetobacter* species. PCR assay resolved by gel electrophoresis showing confirmed *Acinetobacter* spp. targeting the *recA* gene at 425 bp. L = DNA Ladder (100bp); Lane 1 to 9 = Selected *Acinetobacter* isolates; N = Negative control; P = Positive control (*A. baumannii,* DSM Number: 102929).
