# Supplementary material for: Seasonal Shift in Physicochemical Factors Revealed the Ecological Variables that Modulate the Density of Acinetobacter Species in Freshwater Resources
Source: Int J Environ Res Public Health. 2020 May 21;17(10):3606. doi: 10.3390/ijerph17103606 (PMC7277360; doi:10.3390/ijerph17103606)
Supplement: Supplementary file 1 [file ijerph-17-03606-s001.pdf]

Supplementary Materials

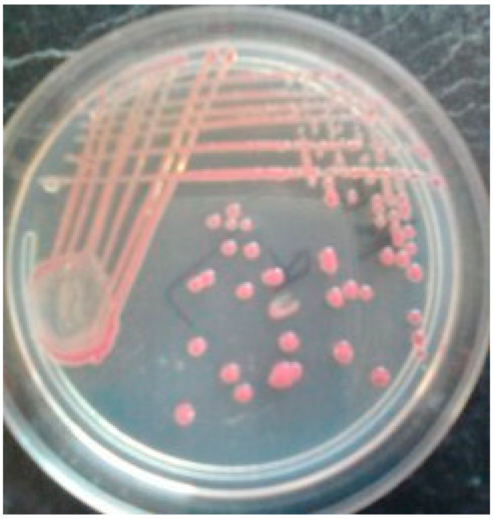

Figure S1. Pure culture of presumptive *Acinetobacter* species streaked on CHROMagar selective medium for the genus *Acinetobacter*: (**A**) Great fish (**B**) Keiskamma and (**C**) Tyhume Rivers. Seasons: Autumn - April and May; Winter - June, July, and August; Spring - September and October; Summer - November, December, January, February, and March.

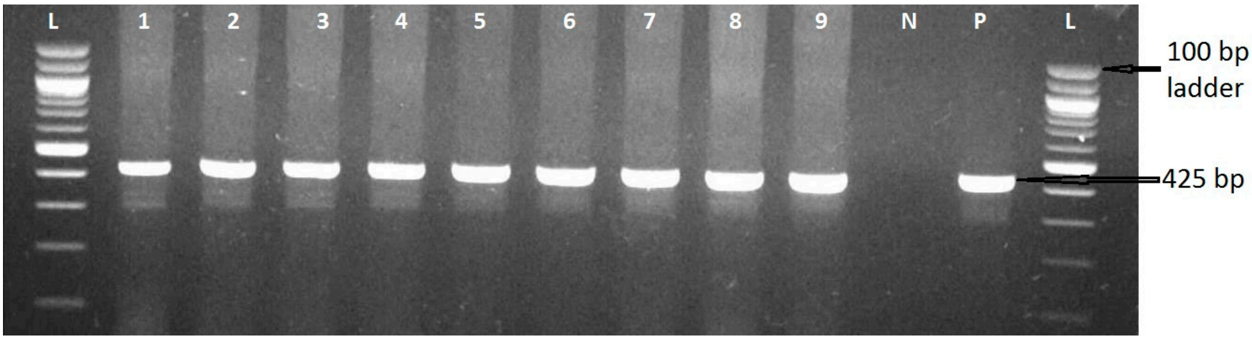

Figure S2. Gel electrophoresis of confirmed *Acinetobacter* species. PCR assay resolved by gel electrophoresis showing confirmed *Acinetobacter* spp. targeting the *recA* gene at 425 bp. L = DNA Ladder (100bp); Lane 1 to 9 = Selected *Acinetobacter* isolates; N = Negative control; P = Positive control (*A. baumannii,* DSM Number: 102929).
